# Upright patient positioning for gantry-free breast radiotherapy: feasibility tests using a robotic chair and specialised bras

**DOI:** 10.3389/fonc.2023.1250678

**Published:** 2023-09-22

**Authors:** Sophie Boisbouvier, Tracy Underwood, Joanna McNamara, Heidi Probst

**Affiliations:** ^1^ Radiation Oncology Department, Centre Léon Bérard, Lyon, France; ^2^ Université Sorbonne Paris Nord, Laboratoire Educations et Promotion de la santé (LEPS), Bobigny, France; ^3^ Research Depatment, Leo Cancer Care Ltd, Horley, United Kingdom; ^4^ Department of Medical Physics and Biomedical Engineering, University College London, London, United Kingdom; ^5^ College of Health, Wellbeing and Life Sciences, Sheffield Hallam University, Sheffield, United Kingdom

**Keywords:** upright radiotherapy, breast radiotherapy, radiotherapy bra, radiotherapy accessibility, patient experience, patient positioning

## Abstract

For external beam radiotherapy using photons or particles, upright patient positioning on a rotating, robotic chair (a gantry-less system) could offer substantial cost savings. In this study, we considered the feasibility of upright breast radiotherapy using a robotic radiotherapy chair, for (i) a cohort of 9 patients who received conventional supine radiotherapy using photons for a diagnosis of primary breast cancer, plus (ii) 7 healthy volunteers, selected to have relatively large bra cup sizes. We studied: overall body positioning, arm positioning, beam access, breast reproducibility, and comfort. Amongst the healthy volunteer cohort, the impact of specialised radiotherapy bras upon inframammary skinfolds (ISF) was also determined, for upright treatment positions. In conclusion, upright body positioning for breast radiotherapy appears to be comfortable and feasible. Of the 9 patients who received conventional, supine radiotherapy (mean age 63.5 years, maximum age 90 years), 7 reported that they preferred upright positioning. Radiotherapy bras were effective in reducing/eliminating ISF for upright body positions, including for very large breasted volunteers. For upright proton radiotherapy to the breast, beam access should be straightforward, even for arms-down treatments, as *en-face* field directions are typically used. For photon radiotherapy, additional research is now required to investigate beam paths and whether, for certain patients, additional immobilisation will be required to keep the contralateral breast free from exposure. Future research should also investigate arm supports custom-designed for upright radiotherapy.

## 1 Introduction

Gantry-free radiotherapy (using fixed treatment beams and rotating, upright patient positioning systems) is attracting interest worldwide as it promises more cost-effective & compact treatment rooms, for all radiation modalities. Volz et al. describe how rotating gantries, and the shielded bunkers that house them, can account for significant portions of proton therapy facility costs: e.g. a proton gantry can cost several million Euros and weigh several hundred tonnes (1). Selecting rotating patient positioning systems over such large gantries could clearly lead to cost savings for particle therapy (1). Even for conventional radiotherapy with photons, gantry-free radiotherapy is being proposed as a means to bring about reductions in room cost, size and shielding (2, 3).

Globally, breast is the most common primary cancer site (4). The optimal radiotherapy utilisation rate for breast carcinoma has been estimated to be 83%, but in clinical practice, actual radiotherapy utilisation rates vary between 24 and 71% (5, 6). A significant barrier to breast radiotherapy utilisation is access to expensive technology (7). Cost-saving innovations such as upright radiotherapy could improve the global accessibility of this treatment.

To date, upright body positioning for breast radiotherapy has not been studied extensively. To our knowledge, the only publication on this topic is a single case report. This documented a morbidly obese patient who exceeded the weight limit for a conventional radiotherapy couch, prompting the clinical team to pursue an upright treatment (8). The patient received external beam radiation to the right breast (in the form of photon tangents) while standing, leaning against the standard couch (8). The team noted that “a specialised chair support (instead of standing) may be of further assistance” (8). Multiple commercial radiotherapy chairs are now entering the market: a historic overview of different chair designs for upright radiotherapy was recently presented by Volz et al. (1).

By itself, a chair is not sufficient to ensure adequate immobilisation, reproducibility, and patient comfort. All aspects of upright patient positioning and the transfer/adaption of immobilisation devices should be studied prior to clinical implementation. For upright body positions, skin folds under the breast (inframammary skin folds (ISFs)) are likely to be more significant, as the breasts will naturally fall vertically down, rather than laterally, as occurs for supine body positioning. Historically, patients with large breast sizes have experienced more acute and late radiotherapy skin toxicities, particularly where their treated breast folds onto surrounding skin (9). In an effort to manage upright inframammary skin folds (ISF) we tested the Chabner XRT bra (with FDA 510K clearance, available commercially) (10) and the SuPPORT 4 All (S4A) bra (under development at Sheffield Hallam University).

Arms-up treatment positions are adopted as standard for supine breast radiotherapy, but are known to be problematic for certain women (11). One small study found that following surgery for breast cancer and routine physiotherapy, 7 out of 30 patients (23%) were unable to maintain a satisfactory ‘arms up’, supine position for breast radiotherapy (12). For most patients, this pose is uncomfortable: one month after breast cancer surgery (around when radiotherapy is likely to begin), >60% of women experience impairments in shoulder flexion and abduction (13). Some patients require intense physiotherapy prior to radiotherapy to regain a sufficient range of shoulder motion, causing delays to their treatment (11). These factors motivated us to also investigate the possibility of arms-down upright treatment set-ups in a robotic chair.

Small-scale published studies suggest that upright radiotherapy may increase comfort for subsets of patients. In the context of head and neck radiotherapy, a sample of 5 patients reported slightly increased comfort for their back and arms, positioned upright relative to supine (14). Further research into upright positioning for a cohort of 16 patients who received conventional, supine radiotherapy to the pelvis, reports an average patient comfort score of 4.1 out of a maximum of 5 (range 3:5) for upright positioning, compared to 3.9 (range 2:5) for supine positioning (15). It has also been hypothesised that patients with obesity, heart problems, superior vena cava obstruction, phrenic nerve injury, dyspnoea, saliva accumulation etc., are likely to find upright radiotherapy more comfortable, as such pre-existing conditions are exacerbated by lying supine (1–3).

The aim of this study was to investigate whether we could position women on a robotic rotating chair, in a manner which would likely be suitable for upright breast RT. Global body positioning, breast positioning, arm positioning, beam access and breast reproducibility were all considered, as were two specialised radiotherapy bras. We also considered comparative comfort associated with upright positioning for a cohort of patients who received conventional, supine radiotherapy to the breast.

## 2 Methods

**Figure 1:**
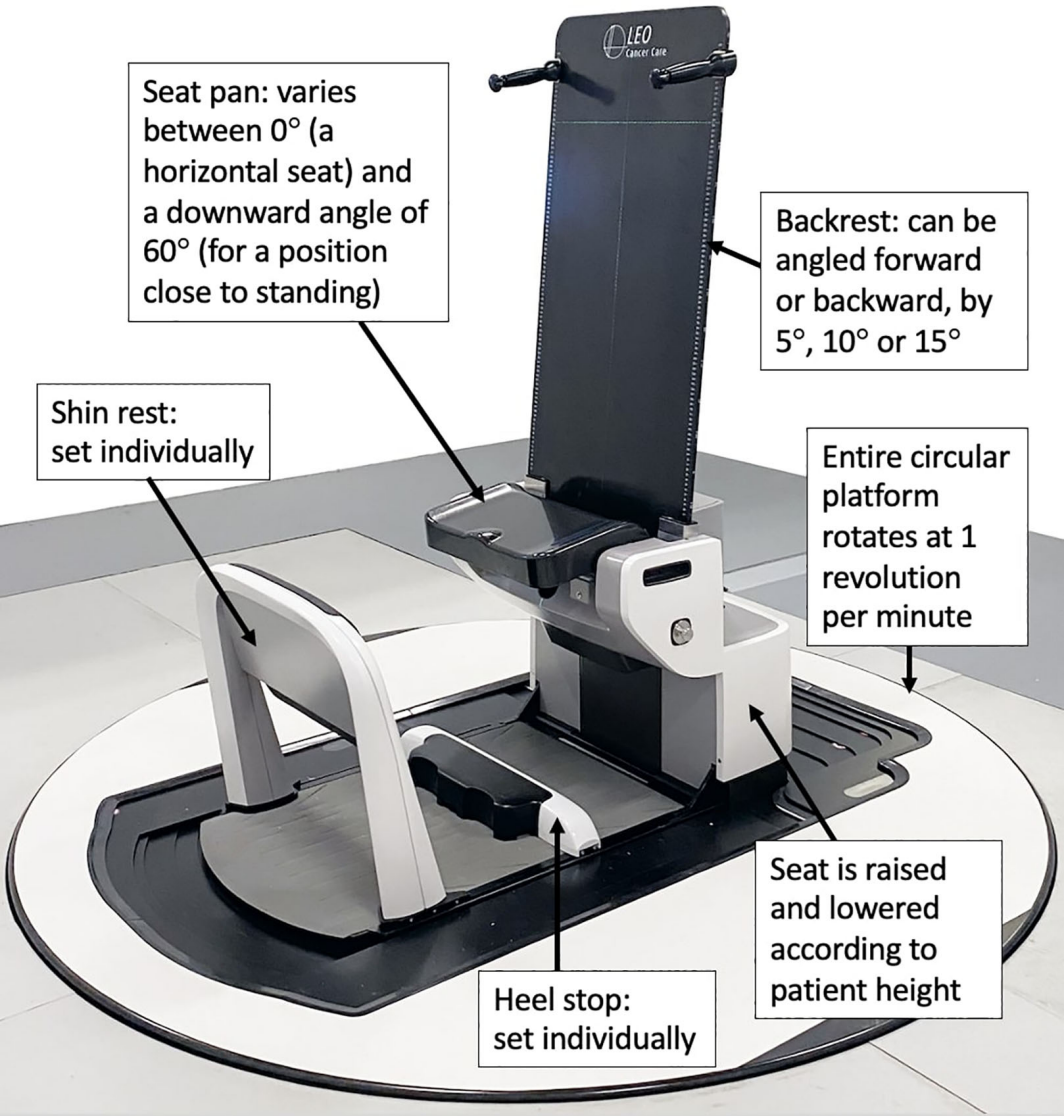
Labelled photograph of the Eve upright patient positioner, courtesy of Leo Cancer Care Ltd. At the time of writing, Leo Cancer Care was in the process of gaining regulatory approval for this device.

The ‘Eve’ upright patient positioner from Leo Cancer Care Ltd (Horley, UK) was utilised^1^. This system consists of a backrest, seat pan, shin rest and heel stop, which can all be adjusted independently (**Figure 1**). The backrest can be angled at 0° (vertical) or forward/backward by 5°, 10° or 15°. The seat pan angle can be varied between 0°C (horizontal, for a sitting position) and 60° (tipped towards the floor, to support a body position that is close to standing). The seat height has a travel range of 47.5 cm. The initial seat pan height is set according to the patient’s lower limb length. The position of the shin rest and the heel stop are also adjusted individually, so that each patient’s lower limbs can be comfortably immobilised, and their body position can be sustained without continuous effort. ’Eve’ rests upon a large circular floor platform capable of 360° rotation (clockwise or counter clockwise, at one revolution per minute). The entire platform can be translated to take the treatment site to the level of the fixed treatment beam.

This was a prospective, proof of concept study. It was conducted in two parts:

**Patient cohort:** global body positioning, arms position, beam access, and comfort were considered for a cohort of 9 patients who received conventional radiotherapy, for a diagnosis of primary breast cancer.
**Healthy volunteer cohort:** comfort, inframammary skin folds (ISFs), specialised bras and topless breast reproducibility were investigated for a cohort of 7 healthy volunteers.

A summary of the two parts of the study is available in **Table 1**.

**Table 1:** A summary of the two cohorts considered and chair set-up methods considered.

	Patient cohort	Healthy volunteer cohort
Sample	2 patients: right-sided breast cancer. 3 patients: left sided breast cancer (one treated using DIBH). 1 patient: right-sided breast cancer with irradiation of the supra- and infraclavicular nodes, internal mammary nodes and axillary lymph nodes. 1 patient: right chest wall plus supra- and infraclavicular lymph nodes. 1 patient: right chest wall plus supra-, infraclavicular and internal mammary lymph nodes. 1 patient: left chest wall, plus supra-, infraclavicular, internal mammary and axillary lymph nodes	7 healthy female volunteers, selected to have relatively large bra cup sizes
Mean age (range)	63.5 years (43 – 90 years)	54.1 (34 – 64 years)
Body Mass Index (BMI) range	19.5 – 43.8	22.2-39.5
Chair set-up methods	Backrest tilted 5° backward Seat pan tilted 50° downwards from horizontal for the standing position, and placed at 0° (horizontal) for the sitting position Vacuum cushion covering the seat pan and the lower part of the backrest No specific immobilisation devices for the arms No specific immobilisation devices for breast	Backrest tilted 10° backward Seat pan tilted downwards from horizontal by 15° Vacuum cushion covering the seat pan and the lower part of the backrest A wing board (Monarch, CIVCO Radiotherapy, USA) for arm support Chabner XRT bra and S4A radiotherapy bras

### 2.1 Recruitment and study methods for the patient cohort

The patient cohort study was conducted at Leon Bérard Center, (Lyon, France) after local institutional review board approval (R201-004-258). Patients who met the following eligibility criteria were invited to participate: (i) referred for radiotherapy for primary breast cancer, with curative intent, (ii) at least 18 years old, (iii) able to give informed consent and (iv) able to walk and stand. In total, nine patients were included. The patients were aged 43- 90 years (mean 63.5 years). Heights ranged from 5’0” to 5’5”, Body Mass Index (BMI) values ranged from 19.5 to 43.8. The treatment received by these patients was unaffected by this research. All received their radiotherapy in the conventional supine position, according to the standard institutional protocol. They were positioned with their arms up for treatment, either using a breast board (Macromedics, The Netherlands) on an Elekta^®^ Linear accelerator or with a T-shape vacuum cushion and the Armshuttle (Qfix, USA) on a Tomotherapy unit (16). In total, 3D conformal radiotherapy was used for 5 patients and Intensity Modulated Radiation Therapy (IMRT) was used for 4 patients, including one patient treated on the Tomotherapy Unit.

For the 9 patients, tests were conducted to assess two positioning variables:

- Global body position (sitting versus standing)
- Arm position (having the arm up on the ipsilateral side, having both arms down along the body, and having both arms behind the body).

**Figure 2:**
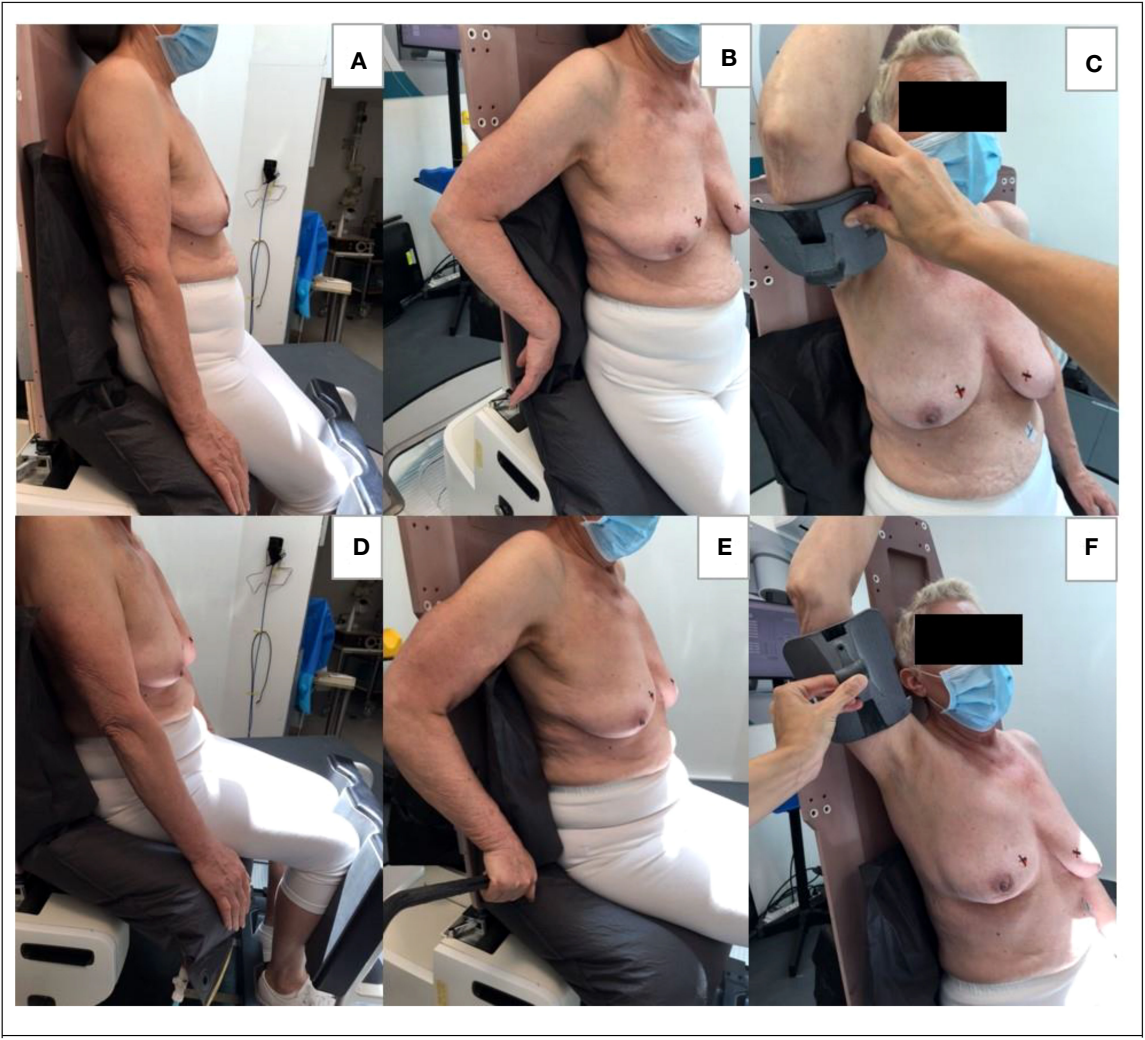
Body positioning for the patient cohort. **(A)** standing position with arms along the body, **(B)** standing position with arms behind the body, **(C)** standing position with one arm up, **(D)** sitting position with arms along the body, **(E)** sitting position with arm behind the body, **(F)** sitting position with one arm up.

These tests were carried out with the ‘Eve’ backrest tilted 5° backward, as this was found to be a comfortable configuration in precursory trials. First, the patients were setup in standing position with the seat pan tipped 50° down and the arms along the body (**Figure 2A**). The shin rest and heel stop were adjusted individually, and individual vacuum cushions (Vac-Lok bags) were moulded, covering Eve’s seat pan & the lower part of the backrest. The radiation therapist then asked the following question: “Globally, what is your level of comfort between 1 (very uncomfortable), and 5 (very comfortable)”. Then, the patient was repositioned with their arms behind their body and the backrest (**Figure 2B**); and with the arm on the treatment side raised, and supported using an arm rest that was held in place by the radiation therapist (**Figure 2C**). Arms-up immobilisation devices specifically for upright radiotherapy are yet to be developed by commercial vendors. As some of the patient cohort presented with limited arm mobility, we choose to raise only the arm on the treatment side, physically supporting it from underneath. For each arm and body position, the question on global patient comfort was repeated. Subsequently, the three arm positions were tested by the patients in a sitting position with the seat pan at 0° (**Figures 2D–F**). For patients treated with 3D conformal radiotherapy, the posterior limit of the field was marked on the skin allowing the radiation therapist to check visually the posterior beam access, particularly for the arms-down body positions. In total the women spent approximately 40 minutes on Eve, with an estimated 5 minutes in each body position. Finally, the patients were questioned about their most preferred position, choosing between sitting, standing and the supine position (the latter as was adopted for their radiotherapy treatment).

### 2.2 Recruitment and study methods for the healthy volunteer cohort

After ethics board review at Sheffield Hallam University (ER37353900), a cohort of 7 healthy women were recruited and gave informed consent to participate in this portion of the study. All were selected to have bra cup-sizes≥C. The women were aged 34 – 64 years (mean 54.1 years). They had heights of 5’2”-5’8”; bra cup sizes C-E; bra band sizes 34-42.” Body Mass Index (BMI) values were 22.2-39.5.

A pre-test session was used to measure the volunteers for both the Chabner XRT bra and S4A radiotherapy bras. On the test days, final fittings and bra selections were performed. Multiple strap configurations are possible for the Chabner XRT bra (for example bringing both straps over one shoulder, or criss-crossing them), but only the conventional, symmetrical strap arrangement was trialed in this study. A series of circular markers were taped to each woman’s bare skin and remained in place throughout the testing (for an example photograph, please see the **Supplementary Material**). Optical cameras were fixed on tripods facing ‘Eve’ and perpendicular to it, enabling quantitative assessment of repositioning uncertainties using the circular markers as reference points on the bare skin. Three repeat positioning tests were performed. In between tests, the volunteers lifted themselves up and off the chair before the therapists repositioned them using room lasers and “tattoo” (pen) marks.

**Figure 3:**
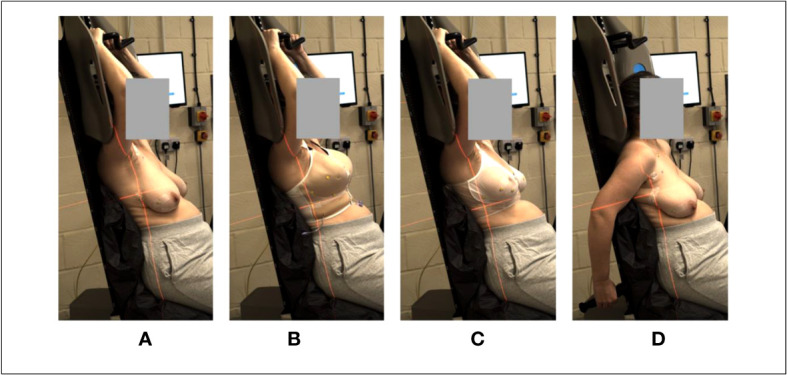
Body positioning for the healthy volunteer cohort. **(A)** arms-up, topless; **(B)** arms-up, S4A bra, **(C)** arms-up Chabner bra XRT, **(D)** arms-down behind the body, topless.

A single Vac-Lok bag was moulded for each woman, covering Eve’s seat pan & the lower part of the backrest. As shown in **Figure 3**, volunteers were asked to trial: both arms-up with/without the treatment bras & an arms-down behind the body position, topless. They were also asked to review sitting on the chair in a natural position (with their arms along their body) for a comfort questionnaire. The arms-up position was implemented using a wing board (Monarch, CIVCO Radiotherapy, USA) attached to the top of the Eve backrest, with a 3D printed support, made in-house. This wing board was designed for conventional, supine radiotherapy and was here repurposed for upright patient positioning. It provided reproducible handle positioning, but no underarm support. All volunteer tests were carried out with the ‘Eve’ backrest tilted 10° backward and the seat pan angled down by 15°. Precursory trials indicated that these settings were likely to reduce abdominal skin folding and bunching of the breasts, particularly for women with large breasts and subcutaneous fat around their abdomens. In total, the volunteers spent ~1 hour on Eve, with ~45mins in the arms-up body position. Measuring tapes were used to determine upright ISF measurements, with & without the bras. Comfort was assessed using questionnaires & 5-point Likert scales.

## 3 Results

### 3.1 Arm and body position and beam clearance

For the 5 patients treated with 3D conformal radiotherapy, based on the posterior limit of the field marked on the skin and the observation of the radiation therapist carrying out the tests, the position with the arms along the body would not allow a treatment with photon tangential fixed beams. With the arms positioned behind the body, treatment with photon tangential fields appeared possible for 3/5 patients. For the remaining 2/5 patients, the backrest prevented the arm from being positioned sufficiently far behind the body: the arm would interfere with a photon posterior tangential fixed beam. For all patients, the position with the ipsilateral arm raised, provided sufficient photon beam access.

**Figure 4:**
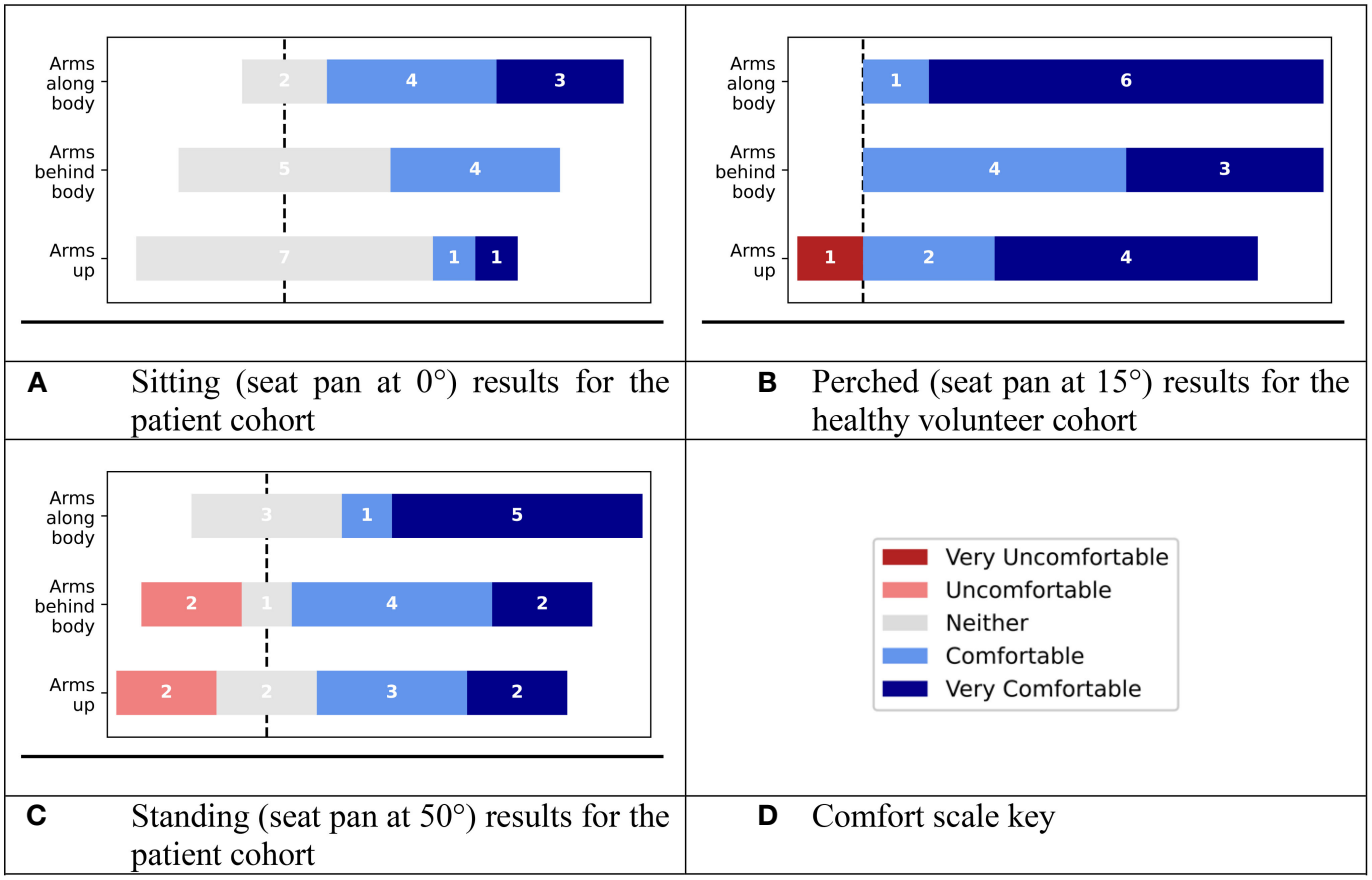
Results of global comfort evaluations, using a 5-part Likert scale for **(A)** sitting position (seat pan at 0°) for the patient cohort, **(B)** perched position (seat pan at 15°) for the healthy volunteer cohort), **(C)** standing position (seat pan at 50°) for the patient cohort. **(D)** Key for the comfort scale.

Results from the comfort questionnaires are shown in **Figure 4**. No patient recorded any variation of the sitting position to be uncomfortable. 2/9 patients recorded the arms-behind and arms-up variations of the standing position to be uncomfortable. Overall, in rank order, the comfort preference for arm positions was: 1) arms posed naturally along the body, 2) arms behind the body, 3) arms-up.

Regarding the most preferred global position for treatment, 4/9 preferred the sitting position and 3/9 preferred the standing position. Finally, 2/9 patients preferred the conventional supine position they adopted for their treatment.

**Figure 5:**
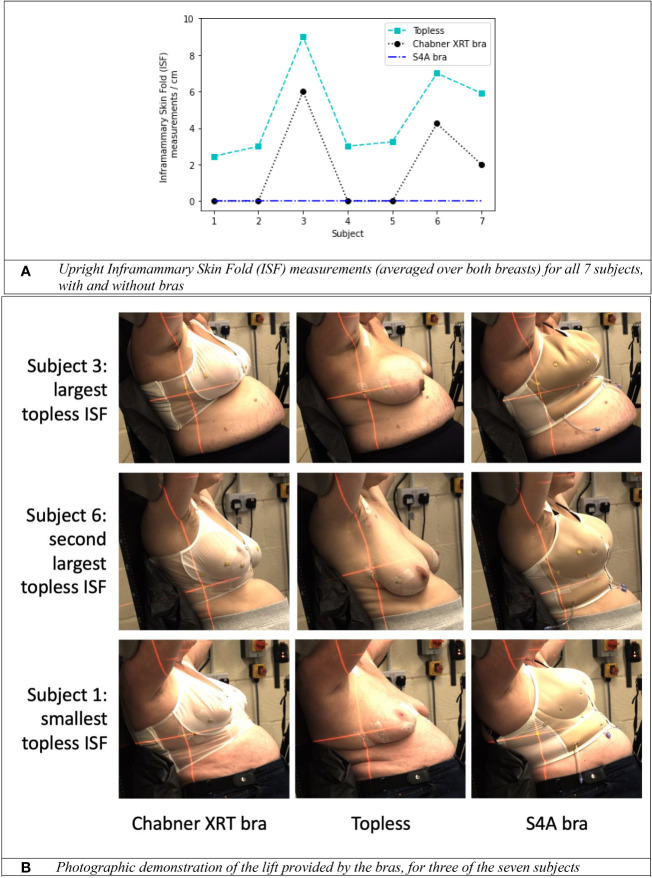
The impact of specialised radiotherapy bras (the Chabner XRT bra and the S4A bra) on upright positioning. **(A)** Inframammary Skin Fold (ISF) measurements (averaged over both breasts) for all 7 subjects, with and without bras. **(B)** Photographic demonstration of the lift provided by the bras, for three of the seven subjects.

In the healthy volunteer sub-study (where participants were selected to have relatively large (≥C) bra cup sizes), tilting Eve’s seat pan down by 15° and tilting the backrest further backwards (to 10°) served to stretch out the body, reducing the extent to which the abdomen bunched against the breasts (**Figure 5**). Among the volunteer cohort, the comfort preference for arm positions followed the same trend as for the patient cohort. All the healthy volunteers reported the arm positions to be comfortable/very comfortable, except one volunteer who found the arms-up position to be ‘very uncomfortable’, due to a pre-existing shoulder condition (adhesive capsulitis, also called ‘frozen shoulder’).

### 3.2 The impact of bras on the inframammary skin fold for upright positioning

For the healthy volunteer cohort, ISF measurement data is presented in **Figure 5A**. With no bra, the mean upright ISF measurement (averaged over both breasts) for the 7 women was 4.8cm (range 2.5cm–9cm). The S4A bra eliminated the ISF for 7/7 women. The Chabner XRT bra eliminated the ISF for 4/7 women. For the 3 most challenging cases (subject 3, subject 6 and subject 7), the Chabner XRT still reduced the skinfold by >3cm in all cases. Both bras were fitted to the best of the researchers’ abilities but it is possible that the ISF could have been reduced for the Chabner XRT, had crossed or tighter strapping arrangements been considered. Photographs for the two women with the largest topless ISF measurements and the woman with the smallest topless ISF measurement are presented as **Figure 5B**. Visibility of underlying tissue, particularly the nipple, was only possible using the translucent Chabner XRT bra (the S4A bra is opaque), but the S4A bra provided more lift. As evident in subject 3 (**Figure 5B**), some fabric bunching occurred for the S4A bra for this participant, this may have been remedied if the internal air pockets that are part of the design of the S4A bra had been inflated for this testing; the air pockets were not inflated for any of the healthy volunteers. Further investigation (e.g. using upright MRI scanning) would be required to determine consistency of underlying breast position in the upright position on a day to day basis.

### 3.3 Comfort assessments for bras and upright positioning

**Figure 6:**
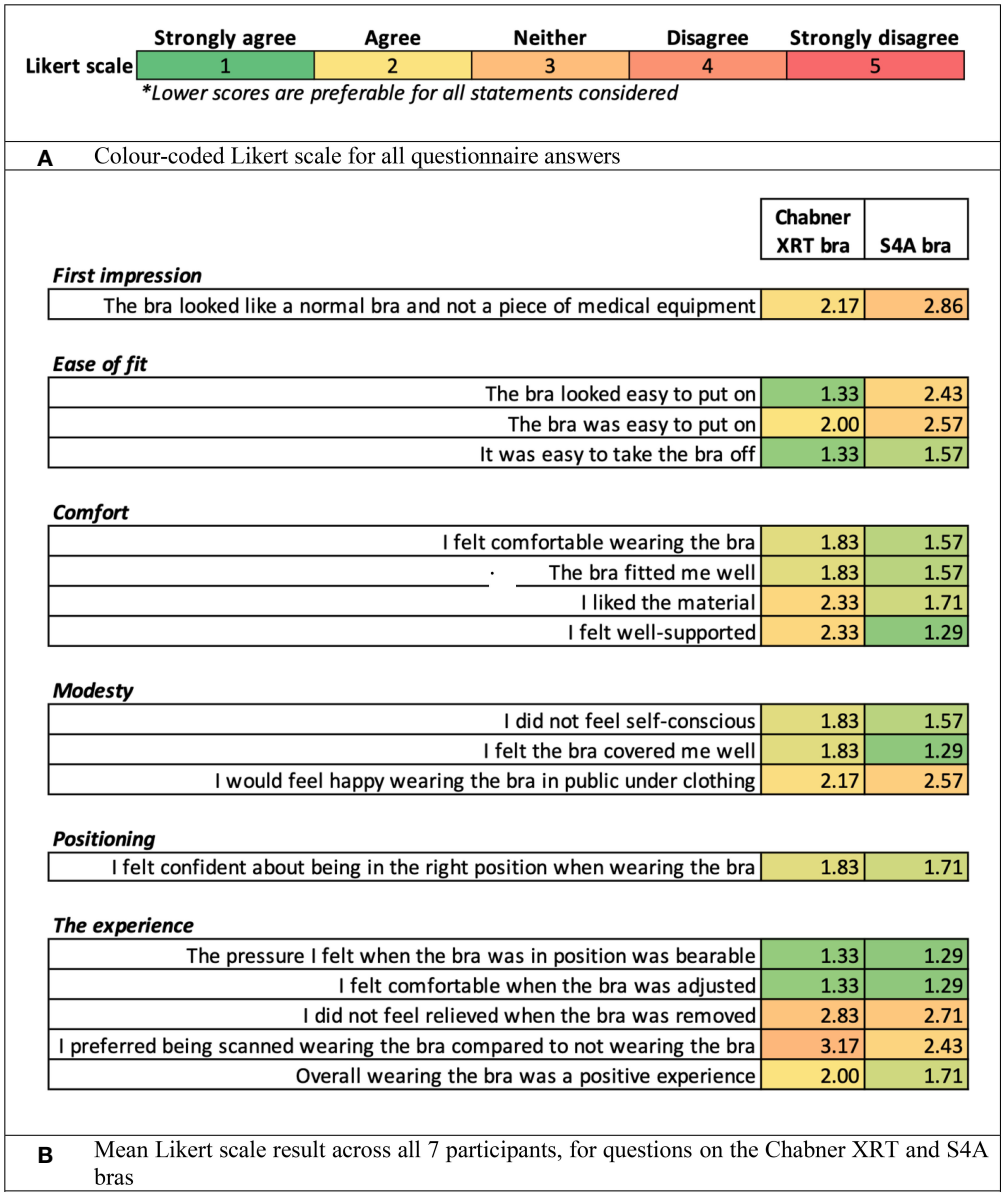
Results from questionnaires considering the healthy volunteers’ views of the upright set-ups and the treatment bras. **(A)** Colour-coded Likert scale for all questionnaire answers. **(B)** Mean Likert scale result across all 7 participants, for questions on the Chabner XRT and S4A bras.

**Figure 6** presents the results from questionnaires which considered the healthy volunteers’ views of the two different radiotherapy bras. Both bras were viewed favourably by the volunteers (**Figure 6B**). The data suggest that the Chabner XRT bra was perceived as being more user-friendly, but the S4A bra was perceived as being more comfortable and well-covering.

### 3.4 Assessments of breast reproducibility

**Figure 7:**
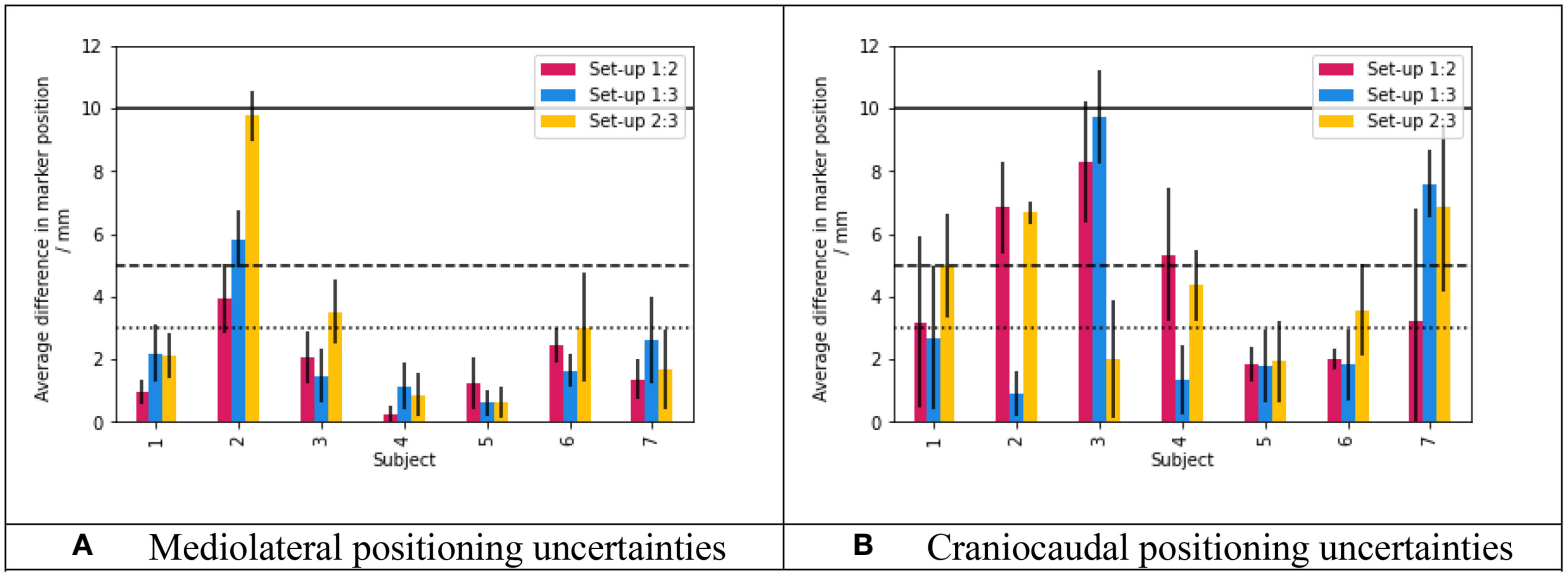
Topless breast re-positioning uncertainties over three repeat set-ups. **(A)** Mediolateral positioning uncertainties. **(B)** Craniocaudal positioning uncertainties. For both plots, data bars show the mean difference in skin marker position between set-ups, across 6 skin markers. The standard deviation of the positional difference across the 6 markers is plotted as the error bar. The red bars show the difference between the first and second set-ups, the blue bars show the difference between the first and third set-ups and finally the yellow bars show the difference between the second and third set-ups. The dotted, dashed and solid horizontal lines indicated differences of 3mm, 5mm and 10mm respectively. Mediolateral and craniocaudal results are presented here as they should represent the extremes in terms of the effects of respiratory motion (mediolateral uncertainties being minimally affected, craniocaudal uncertainties being maximally affected). Anterior-posterior results were quantitatively similar to panel **(A)**.

**Figure 7** shows the average difference in skin marker position introduced when each volunteer was asked to lift themselves off Eve, before the radiation therapists helped them to get back into position. As no form of breathing motion control was used, these quantitative differences in marker position also include effects of respiratory motion: the photographs used in the analysis were obtained at random points within the respiratory cycle. In their comprehensive review, Yoganathan et al. summarise data from X-ray imaging and MRI which consistently indicated greater respiration-induced lung motion in the craniocaudal direction (and, to a lesser extent, the anterior-posterior direction) compared to the mediolateral direction (17). Consequently, of most relevance to the set-up reproducibility (rather than respiratory motion) is the mediolateral uncertainty plot (**Figure 7A**). There were 17/21 repeat set-ups (81%) matched to within 3mm. 19/21 repeat set-ups matched to within 5mm (91%). Results for the anterior-posterior direction were similar to **Figure 7A**). In the craniocaudal direction, a greater impact from respiration-induced motion appears to be present, as expected (**Figure 7B**).

**Figure 8:**
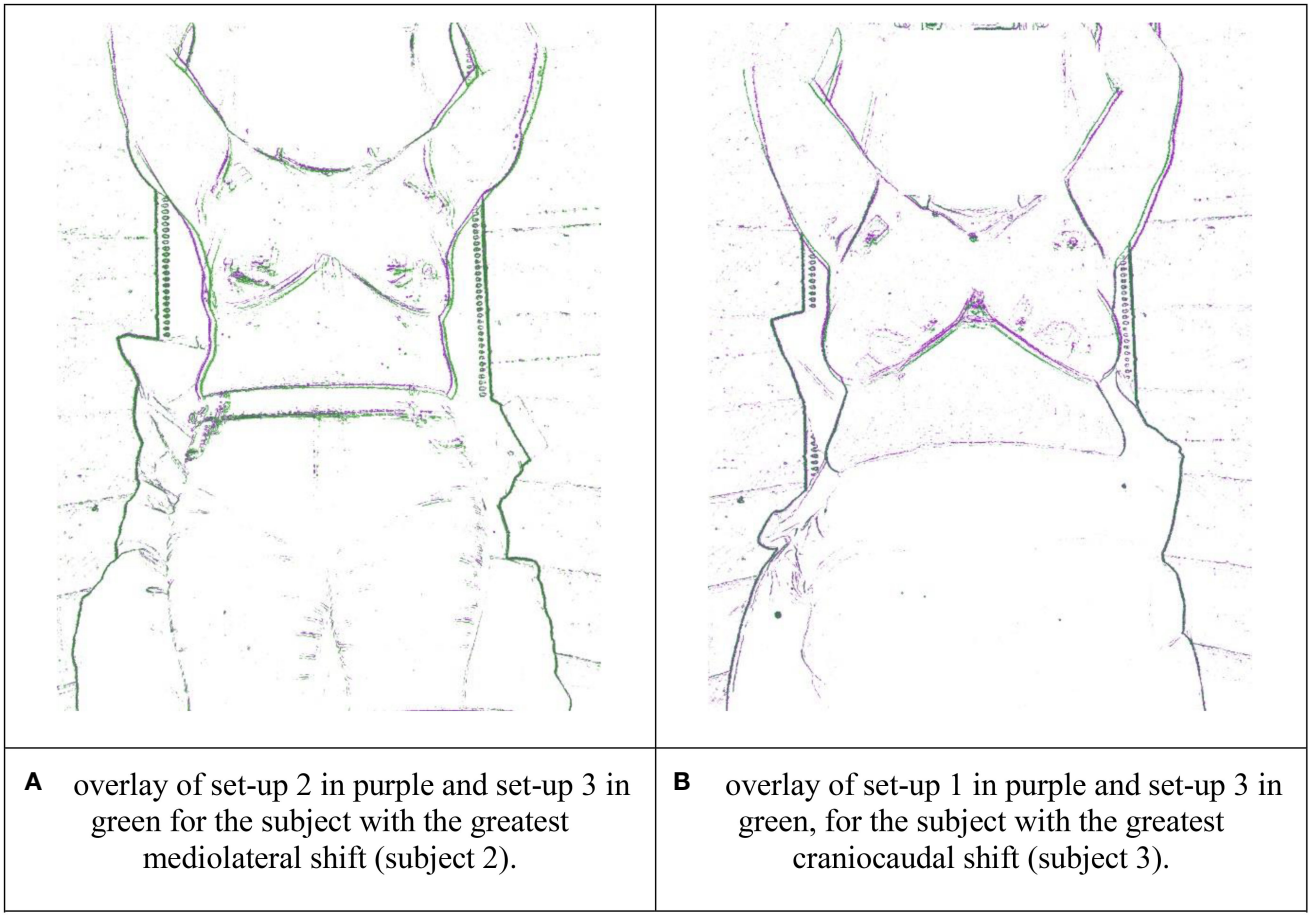
Overlays of the repeat volunteer set-ups with the greatest positional discrepancies in the breast region, with edge detection applied. **(A)** overlay of set-up 2 in purple and set-up 3 in green for the subject with the greatest mediolateral shift (subject 2). **(B)** overlay of set-up 1 in purple and set-up 2 in green, for the subject with the greatest craniocaudal shift (subject 3).

In **Figure 8**, images of two repeated set-ups have been overlaid (after the application of an edge detection algorithm), for the subjects with the greatest mediolateral and craniocaudal shifts between set-ups. These overlays demonstrate that while the lower half of the body was well-secured between repeats, slight shifts were introduced from the waist upwards, including in the positioning of the breasts and the arms. In this case, the arms were positioned using a commercially available wing board designed for supine radiotherapy (Monarch, CIVCO Radiotherapy, USA). This wing board provided a reproducible handle position, rather than any underarm support: it is likely that improvements in arm and breast reproducibility could be realised via arm supports designed specifically for upright radiotherapy of the breast.

## 4 Discussion

We present the first results on upright body positioning using a robotic treatment chair, for breast radiotherapy.

7/9 patients reported that they preferred upright positioning over the supine positioning they experienced for their actual radiotherapy (the mean age of the 9 patients was 63.5 years, the maximum age was 90 years). Amongst the patient cohort there was a slight comfort preference for a seated (seat pan = horizontal, 0°), rather than a standing (seat pan tipped down, 50°) body position. In the volunteer study where participants were selected to have relatively large bra cup sizes, a perched (seat pan tipped down, 15°) body position was adopted. This ‘middle ground’ between seated/standing positions was found to be a good balance between comfort and external anatomy: by slightly angling the seat pan down it was found to be possible to stretch out the abdominal tissue and stomach fat down away from the breasts (and consequently away from the path of the treatment beams). Further work could consider alternative chair settings, but we recommend having the seat pan tipped down by 15° and the backrest tipped back by 5° or 10° as a sensible starting point, which should accommodate women with large breasts and subcutaneous fat around their abdomens. Further control of the abdominal pannus in the seated position (for example using a fabric belt) might also improve geometry.

For all participants within the patient cohort, raising one arm on the ipsilateral side provided adequate beam access for photon tangents. All found it possible to raise their ipsilateral arm, but, as expected, our results suggest that arms down upright treatment positions are likely to be more comfortable. Arms up positioning is known to be complicated by axillary tissue cording (18), general impairments in shoulder flexion and abduction (13), and/or adhesive capsulitis (‘frozen shoulder’) (19), all commonly linked to breast cancer surgery and chemotherapy. According to Yang et al. (19), among breast cancer patients, the risk of adhesive capsulitis is increased by mastectomy and reconstruction after mastectomy. In addition to side-effects from breast cancer treatment, other pre-existing arm and shoulder conditions may render arms-up positioning intolerable for some patients (regardless of whether they are treated supine or upright). Arms down positioning for upright breast radiotherapy needs further investigation, however, particularly with regard to beam access for photon tangential beams. In our study, treatments with the arms down behind the standard ‘Eve’ backrest appeared to provide sufficient photon beam access (in terms of the posterior edge of the 3D conformal field avoiding the ipsilateral arm) for 3/5 patients. It is possible that alternative ‘arms-down’ back rest designs, with cut-away sections for the arms, could offer sufficient photon beam access for a greater proportion of patients. Further work is also required to consider the advantageous manipulation of the contralateral breast for upright positioning and to reduce ‘dose splash’ from photon tangents. For example, the S4A bra includes inflatable air pockets which can be filled with air using a syringe, to push contralateral breast tissue laterally out of photon treatment fields (a diagram of the S4A bra is included as **Supplementary Material**, the air pockets were not filled in this study). The Chabner XRT bra might be combined with an additional strap to manipulate the contralateral breast. For breast radiotherapy using protons, it is anticipated that upright body positions with the arms down behind the backrest would provide suitable beam access, as en-face field directions (rather than tangents) are used. For this reason, proton dose to the contra-lateral breast should be minimal, even for upright body positions. Arms down proton treatments for breast radiotherapy have previously been implemented for supine body positions, as described by Depauw et al. (11).

For the volunteer cohort of 7 healthy women selected to have relatively large bra cup sizes, ISFs were in some cases substantial (range 2.5cm–9cm) for topless upright set-ups. Two specialised radiotherapy bras were effective in minimising these skin folds and were viewed favourably by the volunteers in terms of comfort. The S4A bra provided more substantial lift and eliminated the ISF for all participants. For photon radiotherapy, because a bra might introduce a bolus effect: surface dose testing, as performed for both bras (unpublished work) must be completed to determine the bra safe. Of the two bras tested here, the Chabner XRT bra was thinner: visibility of the underlying breast was maintained for this bra. For the Chabner XRT bra, a preliminary study reported no acute skin toxicities > grade 2 and no observed differences with patients treated without a bra (20). Similarly, in a randomised clinical feasibility study of the S4A bra, Radiation Therapy Oncology Group (RTOG) skin toxicity scores were comparable between the two groups (with and without the bra), and no grade 3 reactions were reported in either arm (21). The Chabner XRT bra is commercially available (CIVCO Radiotherapy, Chabner XRT) whereas the S4A bra is still under development at Sheffield Hallam University.

Overlays of repeat volunteer set-up images (e.g. **Figure 8**) echoed the findings of Boisbouvier et al. (15), where positioning of the lower body was found to be consistently reproducible when ‘Eve’ was used with personalised vacuum cushions. As shown in **Figure 8**, discrepancies in repeat positioning increased further up the body, through the torso, the breasts and the arms. Mediolaterally, 17/21 of volunteer repeat set-ups (81%) matched to within 3 mm in the breast region. Further research to verify and improve set-up reproducibility is warranted. For example, there is considerable scope to improve upper body reproducibility and stability through the development of arm supports designed specifically for upright radiotherapy. One study has considered the application of “soft robotics” for this purpose (22). The magnitude of respiratory motion for upright body positioning should also be considered, as may the application of respiratory motion-management strategies. In this study, the therapists repositioned the volunteers using room lasers and “tattoo” (pen) marks only: optical camera images/surface guidance methods were not used to guide volunteer repositioning. It is well known that using surface guidance can improve the accuracy of supine patient set-ups for breast radiotherapy (23): in principle, such techniques should bring similar benefits to upright radiotherapy set-ups also.

Limitations of this study include the following. Firstly, only 9 patients and 7 healthy volunteers were enrolled. However, at the development phase of an innovation, small sample studies can play a key role (24) and despite its cost saving potential, only a single patient case-study on upright radiotherapy to the breast has been published to date (8). It is also possible that the raising/lowering of a CT scanner over an upright patient may impact that person’s psychological comfort: further investigation is warranted on this topic. Finally, our study evaluated breast reproducibility based on camera images. Ultimately repeat vertical CT scans are required to verify both the reproducibility of the internal anatomy, and upright dose distributions/clinical treatment quality. To-date no full treatment planning study has been published comparing radiotherapy dosimetry upright and supine, for any tumour site. This will be a key area for future research as upright CT scans become available. On average, lung volumes are greater for sitting and standing body positions compared to supine body positions (25, 26): it has been hypothesised that greater lung-sparing and organ at risk -sparing may be possible for upright radiotherapy of the breast (3).

## 5 Conclusion

Upright body positioning for breast radiotherapy appears to be comfortable and feasible. 7/9 patients who received conventional, supine radiotherapy for a diagnosis of primary breast cancer (mean age 63.5 years, maximum age 90 years) reported that they preferred upright positioning. Even for larger breasted women, radiotherapy bras were effective in reducing/eliminating ISF. For proton radiotherapy, beam access should be straightforward as en-face field directions are typically used. For photon radiotherapy, additional research is now warranted to investigate beam paths and whether, for certain patients, additional immobilisation will be required to keep the contralateral breast out of the treatment fields. Upright body positioning may provide additional flexibility in arm positioning, potentially enabling patients’ lowered arms to be moved behind the breasts and the body, away from the treatment fields. Further research is ongoing into (1) reducing set-up errors by further stabilising the upper body (e.g. through custom-designed arm supports), (2) photon beam access, (3) internal anatomy and (4) patient perspectives of upright positioning.

## Data availability statement

The original contributions presented in the study are included in the article/**Supplementary Material**. Further inquiries can be directed to the corresponding author, Sophie Boisbouvier, sophie.boisbouvier@lyon.unicancer.fr

## Ethics statement

The studies involving humans were approved by Ethics board review at Sheffield Hallam University (ER37353900 for the healthy volunteers study) and the local institutional review board approval (R201-004-258) at Leon Bérard Centre for the study of patients. The studies were conducted in accordance with the local legislation and institutional requirements. The participants provided their written informed consent to participate in this study.

## Author contributions

SB: design, acquisition and analysis of patient cohort study; drafting of the manuscript; TU: design, acquisition and analysis of volunteer cohort study; drafting of the manuscript; JM: design and data acquisition of volunteer cohort study; critical revision of the manuscript; HP: design, data acquisition and analysis of volunteer cohort study; critical revision of the manuscript. All authors contributed to the article and approved the submitted version.

## Footnotes

1 At the time of writing, Leo Cancer Care was in the process of gaining regulatory approval for this device.
