# Supplementary material for: Upright patient positioning for gantry-free breast radiotherapy: feasibility tests using a robotic chair and specialised bras
Source: Front Oncol. 2023 Sep 22;13:1250678. doi: 10.3389/fonc.2023.1250678 (PMC10556698; doi:10.3389/fonc.2023.1250678)
Supplement: Supplementary file 1 [file DataSheet_1.docx]

Supplementary Material

Uprightpatientpositioningfor gantry-freebreastradiotherapy: feasibilitytestsusingarobotic chairandspecialisedbrasArticle Title

Sophie Boisbouvier ^1,2*†^, TracyUnderwood^3,4*†^, Joanna McNamara, Heidi Probst

*** Correspondence:** SophieBoisbouvier: [sophie.boisbouvier@lyon.unicancer.fr](mailto:sophie.boisbouvier@lyon.unicancer.fr)

^1^Radiation Oncology Department, Centre Le´on Be´rard, Lyon, France

^2^Universite´ Sorbonne Paris Nord, Laboratoire Educations et Promotion de la sante´ (LEPS), Bobigny, France

^3^Leo Cancer Care Ltd, Horley, United Kingdom

^4^Department of Medical Physics and Biomedical Engineering, University College London, London, United Kingdom,

5College of Health, Well-being and Life Sciences, Sheffield Hallam University, Sheffield, United Kingdom

# Supplementary Figures


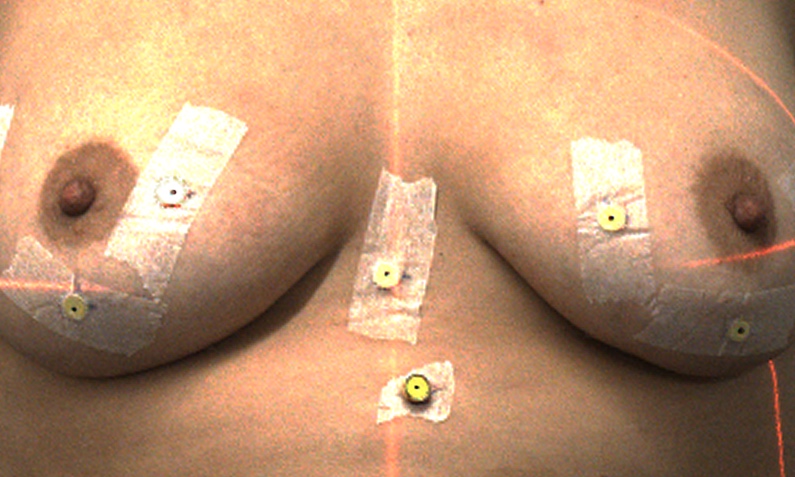


**Supplementary Figure 1.** Example of circular markers taped to the topless breast. These markers were used to assess the reproducibility of repeat set-ups, using optical camera images.


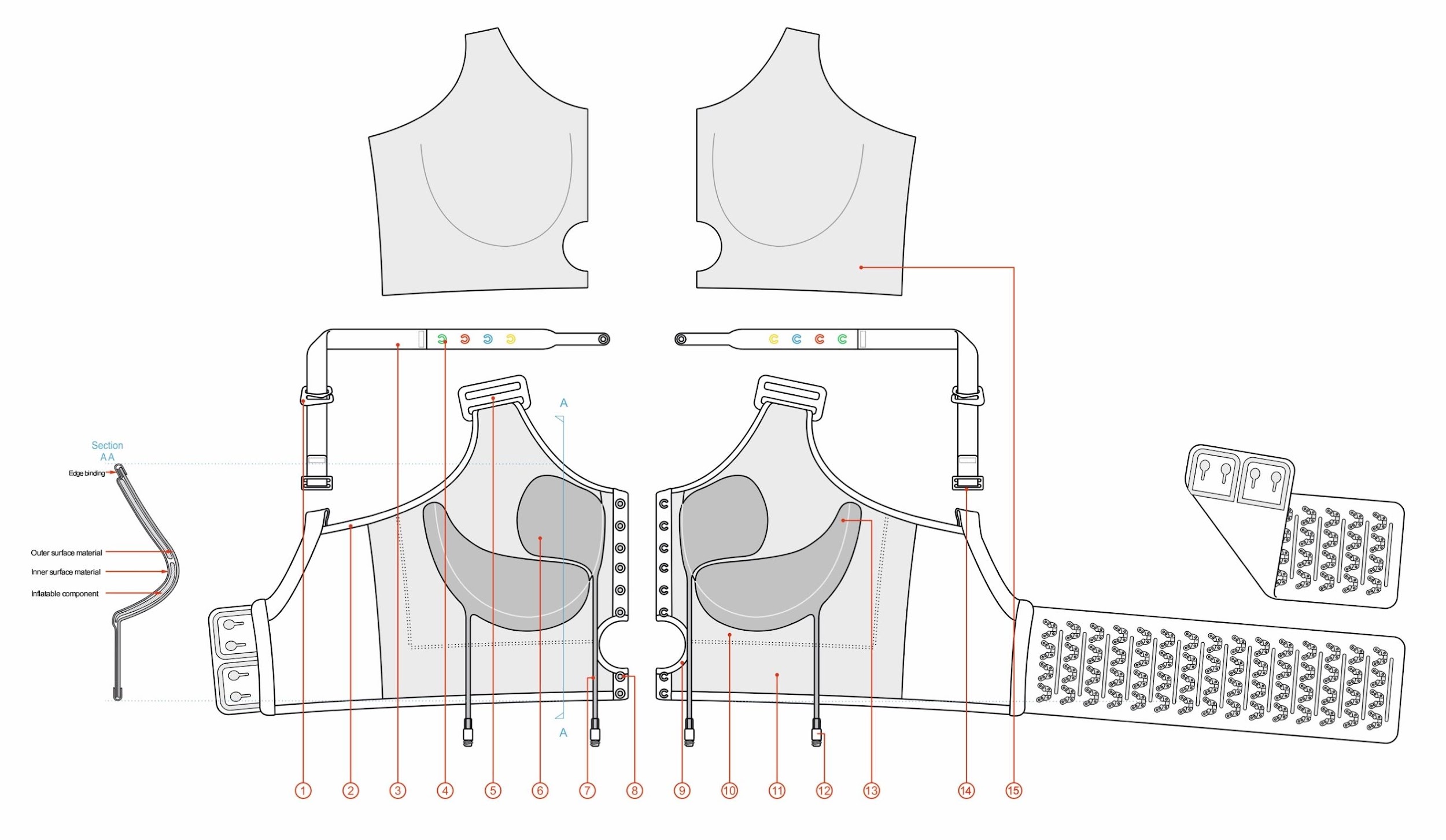


###
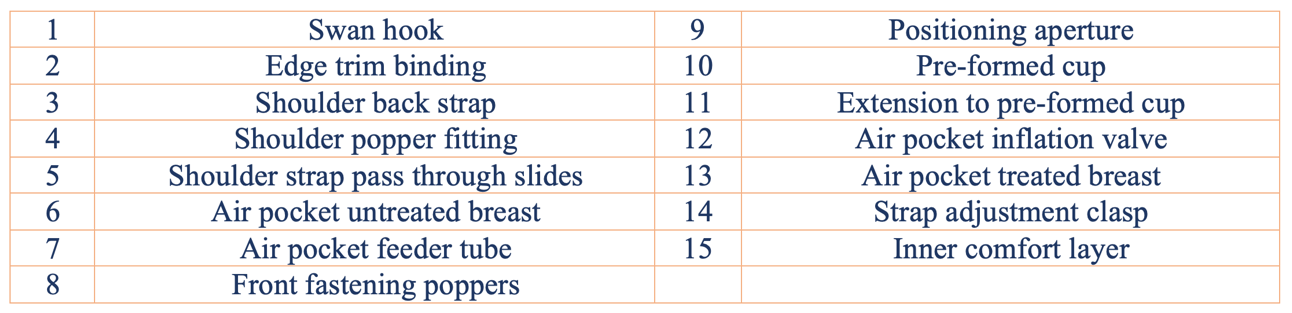


**Supplementary Figure 2.** A labelled schematic of the SuPPORT 4 All Bra.
